# Combination therapy in cancer: effects of angiogenesis inhibitors on drug pharmacokinetics and pharmacodynamics

**DOI:** 10.1186/s40880-016-0123-1

**Published:** 2016-06-29

**Authors:** Ilaria Fuso Nerini, Marta Cesca, Francesca Bizzaro, Raffaella Giavazzi

**Affiliations:** Department of Oncology, IRCCS-Mario Negri Institute for Pharmacological Research, Via La Masa 19, 20156 Milan, Italy

**Keywords:** Combination therapies, Angiogenesis inhibitors, Drug delivery, Tumor microenvironment

## Abstract

Validated preclinical studies have provided evidence that anti-vascular endothelial growth factor (VEGF) compounds enhance the activity of subsequent antitumor therapy, but the mechanism of this potentiation is far from clear. The most widespread explanation is enhanced delivery of therapeutics due to vascular remodeling, lower interstitial pressure, and increased blood flow. While the antiangiogenic effects on vascular morphology have been fairly consistent in both preclinical and clinical settings, the improvement of tumor vessel function is debated. This review focuses on the effect of anti-VEGF therapy on tumor microenvironment morphology and functions, and its therapeutic benefits when combined with other therapies. The uptake and spatial distribution of chemotherapeutic agents into the tumor after anti-VEGF are examined.

## Background

Deregulation of angiogenesis is a hallmark of cancer which causes an abnormal microenvironment, promoting tumor progression and affecting the delivery of chemo-, radio-, and immunotherapy [1]. Tumor angiogenesis offers an attractive therapeutic target, shared by most cancers. Angiogenesis inhibitors have therefore been investigated and exploited for their therapeutic application in most human tumors. The most validated antiangiogenic approaches act on the vascular endothelial growth factor (VEGF) axis, blocking VEGF or its receptors (VEGFRs) [2, 3]. Examples include the humanized anti-VEGF monoclonal antibody bevacizumab, approved in the clinic for a number of malignancies [4], and the VEGF-Trap protein aflibercept formed by the fusion of the immunoglobulin domain of VEGFR with the human IgG Fc fragment, approved for second-line treatment of metastatic colorectal cancer (CRC) [5]; both compounds are in general used in combination with chemotherapy. A number of small molecules, such as sunitinib, sorafenib, pazopanib, and cediranib, inhibit the tyrosine kinase activity of VEGFR and have been approved as single therapies [6, 7]; due to frequent severe adverse effects, they are rarely used in combination with chemotherapy.

Because of the structural similarities between VEGFR and other receptor tyrosine kinases (RTKs), these receptor tyrosine kinase inhibitors (RTKIs) often inhibit multiple targets, thus affecting not only the tumor vasculature but other components of the tumor stroma and neoplastic cells themselves. On the other hand, some drugs that were developed for their cytotoxic effects on tumor cells also induce antiangiogenic responses by down-regulating pro-angiogenic factors or by directly targeting endothelial cells. For example, paclitaxel and other tubulin-binding agents target tumor vessels, inhibiting endothelial cell functions related to angiogenesis, at lower concentrations than those required for the anti-mitotic activity [8].

We review therapeutic strategies that use antiangiogenics in combination with chemotherapy to enhance their potential. We focus on anti-VEGF compounds, particularly bevacizumab, since they are the main angiogenesis inhibitors used in clinical application combined with chemotherapy. We illustrate the antitumor effects of the combination, taking into account the effects of VEGF inhibition on pharmacokinetics, biodistribution, and tumor penetration of chemotherapy.

## Pharmacodynamic and functional effects of antiangiogenics on tumor microenvironment

The vasculature network in tumors is structurally abnormal, with tortuousities and dilatations, disproportionate branching, and arteriovenous shunts. The angiogenic process, driven by high levels of growth factors and inflammatory cytokines, is extensive, and the newly formed vessels are extremely leaky due to defective pericyte coverage and discontinuous basement membranes. The structural abnormalities of the tumor vasculature, together with the compression of blood vessels by cancer cells, cause functional impairment of blood flow, such as temporary stagnation, turbulent flow, local hemorrhages, and high outflow of plasma macromolecules. The consequences are increased tumor interstitial fluid pressure (TIFP) and the formation of hypoxic/necrotic regions [9]. These structural and functional aberrations occur in a heterogeneous spatial (in different areas of tumor tissue) and temporal (during tumor growth and progression) manner.

Antiangiogenic therapy restores the balance between pro- and antiangiogenic molecules and the vascular architecture of tumor tissue by pruning immature vessels and remodeling the remaining ones [10, 11]. Several preclinical studies agree on the modifications induced by antiangiogenics (e.g. bevacizumab) in tumor vasculature, both macroscopically and microscopically. In different tumor models, antiangiogenic treatment reduced the density, diameter, and tortuosity of capillaries and induced vessel maturation by re-establishing pericyte and basement membrane coverage [12].

Clinical studies have provided evidence of vascular changes in cancer patients treated with antiangiogenic agents, despite the difficulties of biopsying tumors after pharmacologic therapy [13–17]. Tumor biopsies from non-metastatic CRC patients from a phase I study showed evidence of the antiangiogenic effects of anti-VEGF therapy with bevacizumab [18]. Pathologic analysis indicated that bevacizumab reduced tumors from hyperemic, hemorrhagic lesions to pale masses, and microscopically led to an increase in pericyte coverage, favoring vessel maturation and stabilization [18].

Whether these morphological changes are accompanied by functional modifications of the tumor vasculature is debated. Hypothetically, antiangiogenics can improve blood perfusion, with a consequent drop in TIFP and alleviation of hypoxia [11, 12]. This suggests that the blood transport capacity of vessels that survive antiangiogenic treatment is increased, favoring the arrival of oxygen and nutrients to tumor tissues. If anti-VEGF therapy really improves the blood flow and oxygenation of tumor tissue, then the question arises whether it may actually favor tumor growth. Although this is possible in theory, there are no studies describing promotion of tumor growth with antiangiogenics, rather the contrary is true. This is probably due to the long-term inhibition of new vessel formation that starves the tumor after a temporary favorable increase in blood flow and oxygenation.

In preclinical studies, bevacizumab reduced the tumor uptake of fluorescent dyes—analyzed by microscopy, or of contrast agents—seen with vital imaging techniques [19–21]. Using intravital multiphoton microscopy to monitor the transit velocity of erythrocytes, it was seen that bevacizumab increased blood flow velocity and reduced vascular permeability in the tumor [22]. Arterial spin labeling magnetic resonance imaging (ASL-MRI) showed a specific decrease of tumor perfusion after bevacizumab, which was associated with the reduction of vessel diameter observed with histological analysis [23]. Clinical studies reported reduced penetration of contrast agents in the neoplastic bulk, independently of tumor shrinkage [24–29]. These effects are probably caused by vessel constriction, due to the suppression of nitric oxide production by bevacizumab [30], and/or the reduction of vessel permeability because of VEGF sequestration [31]. Changes in tumor perfusion after angiogenesis inhibition can have predictive value in patients. Glioblastoma patients with increased tumor blood perfusion are those most likely to benefit from antiangiogenic treatment [32].

Evaluating downstream effects of antiangiogenics on tumor tissue is difficult. Reliable methods to measure TIFP are still lacking. Wick-in-needle (WIN), which is the best standard to quantify TIFP, is limited by its invasiveness causing tissue damage that makes measurement untrustworthy [33]. Despite this problem, several experimental studies have reported decreases in TIFP after treatment with VEGF antagonists [9, 18, 20, 34].

Contrasting results were obtained regarding the effects of angiogenesis inhibitors on tumor hypoxia. The different outcomes can be explained by the kinetics of the pharmacodynamic effects of antiangiogenics. The improvement in tumor oxygenation seems to last 2–4 days after anti-VEGF treatment [11]. At later times, increased intratumoral hypoxia was reported after bevacizumab treatment, probably due to the reduction of vessel density [35]. The transient drop in tumor hypoxia can be exploited to enhance the efficacy of radiotherapy [36]. There is also preclinical evidence that combining antiangiogenic agents with immunotherapy can improve the response, probably due to a reduction in tumor tissue hypoxia that favors the delivery of immune effector cells [37].

## Antiangiogenesis in combination with chemotherapy improves anticancer efficacy

Preclinical studies reported responses in different tumor models after treatment with anti-VEGF monoclonal antibodies [12], and these were significantly better when combined with other treatment modalities, mainly chemotherapy [20, 34, 38–40]. In the clinical setting, anti-VEGF has been proven effective as monotherapy only in certain cancers [41], but, added to first-line chemotherapy, it significantly improved clinical outcomes in different malignancies [42–45]. Bevacizumab has now been approved in combination with standard chemotherapy for patients with metastatic CRC [46], recurrent/advanced non-small cell lung cancer (NSCLC) [47], advanced cervical cancer [48], and advanced ovarian cancer [49–52], although its effectiveness is limited and lower than expected, especially in end-stage tumors. In general, angiogenesis inhibitors, and bevacizumab in particular, can be administered for extended periods safely and with manageable toxicity, so the potential benefit of the treatment is not limited by increased adverse events.

Several explanations have been proposed for the mechanisms by which antiangiogenic agents boost the efficacy of chemotherapy: (a) a direct effect on neoplastic cell viability and induction of cytotoxicity independently of the vascular effects [22]; (b) impairment of the tumor cell’s ability to repopulate between successive courses of chemotherapy [53]; (c) block of pro-survival signals and consequent chemosensitizing effect on endothelial cells, leading to disruption of vessels and starvation of neoplastic cells [38]; (d) “normalization” of the vascular microenvironment causing TIFP to drop and increasing intratumoral delivery of chemotherapy [54, 55]; (e) temporary improvement of the oxygen and nutrient supply to tumor cells that renders them more sensitive to the cytotoxic activity [56]; (f) killing or inhibiting the mobilization of pro-angiogenic bone marrow-derived circulating endothelial progenitors [57]; (g) stimulation of the host immune response against the tumor by improving tumor delivery of immune cells and/or alleviating the tumor immunosuppressive microenvironment [58].

Alarmingly, some preclinical studies reported increased dissemination and metastases after VEGF/VEGFR inhibition, mainly with RTKIs [59–61] and in a neoadjuvant setting or when distant metastases are not established yet [62]. This seems to be counteracted by an appropriate combination with certain cytotoxic drugs [62]. Experimental evidence of increased invasion and metastasis after bevacizumab is not consistent [40, 63]. Accordingly, clinical trials have not yet reported any increase in malignancy on VEGF inhibition [64], probably because some antiangiogenics are used in an adjuvant setting (e.g., RTKIs) and/or in combination with chemotherapy (e.g., bevacizumab). Several trials indicate that prolonged progression-free survival and improved response rates after antiangiogenic therapy are not always translated into an overall survival benefit [4, 65].

Optimization of the treatment schedule combining antiangiogenics and cytotoxics is becoming increasingly important to achieve efficacy. Preclinical and clinical evidence indicates that the benefit of angiogenesis inhibition is transient, and there is only a narrow window of opportunity during when synergy with chemotherapy can be achieved [11]. The optimal dosing of the antiangiogenic agent is also critical, as excessive suppression of the tumor vasculature may be counterproductive [37]. Improved clinical responses have been observed when chemotherapy was combined with low- rather than high-dose bevacizumab [66].

## Effects of antiangiogenics on pharmacokinetics and tumor uptake of chemotherapy

In terms of drug distribution, one would expect the vascular access of anticancer drugs to tumors to be impaired by inhibiting angiogenesis. However, the enhanced response to chemotherapeutics when given in combination with antiangiogenic compounds suggests that they do not necessarily reduce drug delivery to tumor tissue, but rather the opposite. The “normalization” theory provides an explanation for this apparent paradox, according to which an appropriate dose of antiangiogenic agent can restore normal blood flow and reduce TIFP, thus favoring the penetration of cytotoxic agents [11].

Although the effects of antiangiogenic therapy on the remodeling of vascular architecture have been demonstrated in various preclinical models, the consequences on drug distribution are often under-explored. Table 1 summarizes some in vivo experimental studies in which anti-VEGF antibodies were administered prior to antitumor agents and concentrations of the second drug in the tumor were measured with different methods. It is hard to compare the results because of the different tumor models and histotypes, the angiogenesis inhibitor dosages, and the combination schedules. Most studies agree on the morphological vessel changes after anti-VEGF treatment, such as decreased vessel density and greater pericyte coverage, but this is not always associated with functional modifications, such as increases in vessel perfusion and permeability. Results are discordant in terms of drug delivery to the tumor: some studies show increased uptake after antiangiogenic therapy [20, 34, 67–71], but others report reduced drug delivery [72–77]. Enhanced tumor uptake of chemotherapeutics was concomitant in some instances with improvement of vessel functionality [68, 78], whereas in other cases there was worsening of perfusion/permeability [34, 69]. Inefficient drug delivery was often associated with the reduction of tumor perfusion or vessel permeability.

**Table 1:** Preclinical studies on combinations of anti-vascular endothelial growth factor (VEGF) drugs with conventional therapies, or other therapeutics

Tumor model		Drugs		Treatment schedule		Effects of AI		ST measurement	Ref.
Name	Type	Angiogenesis inhibitor (AI)	Subsequent treatment (ST)	Time of AI before ST	Time of biopsy after ST	Tumor vasculature	Tumor drug uptake		
NB-1691	Neuroblastoma	Bevacizumab ≈ 8 mg/kg	Topotecan, etoposide	−7 days	1 h	↓ vessel density ↓ permeability ↓ necrosis	= topotecan	HPLC	[20]
				−3 days		↓ vessel density ↓ permeability ↑ perfusion	↑ topotecan =/↑ etoposide		
				−1 day		↓ vessel density ↓ permeability ↑ perfusion	=/↑ topotecan ↑ etoposide		
				0 day			= topotecan = etoposide		
SK-N-AS	Neuroblastoma	Bevacizumab ≈ 8 mg/kg	Topotecan	−7 days	1 h		=/↑ topotecan		
				−3 days			↑ topotecan		
				−1 day			= topotecan		
				0 day			= topotecan		
DM443	Melanoma	Bevacizumab 5 mg/kg	Melphalan	−3 days	1 day	↓ perfusion/permeability = vessel density	↑ melphalan	IHC of DNA-melphalan adduct	[34]
DM738	Melanoma	Bevacizumab 5 mg/kg	Melphalan	−3 days	1 day	↓ perfusion/permeability = vessel density	↑ melphalan		
SAS	Head and neck cancer	Bevacizumab ≈ 10 mg/kg	BPA	−7 days	1 h		= BPA	PGA	[66]
				−3 days		↓ vessel density	= BPA		
				−1 day		↑ perfusion/permeability	↑ BPA		
				−0.5 day			↑ BPA		
HCCLM3	Hepatic cancer	Bevacizumab 5 mg/kg	Doxorubicin	−7 days	1 h	↓ vessel density	= doxorubicin	HPLC	[67]
				−5 days			↑ doxorubicin		
				−3 days			↑ doxorubicin		
				−1 day			= doxorubicin		
				0 day			= doxorubicin		
MX-1	Breast cancer	Bevacizumab 5 mg/kg	Paclitaxel	−1 h	2 days	↓ perfusion/permeability	↑ paclitaxel	HPLC	[68]
A549	Lung cancer	Bevacizumab 5 mg/kg	Paclitaxel	−1 h	2 days		↑ paclitaxel		
OSC-19	Oral squamos cancer	Bevacizumab ≈ 4 mg/kg	Cetuximab	−3 days	From 1 to 13 days	↑ pericyte coverage	↑ cetuximab	FM	[70]
SCC-1	Oral squamos cancer	Bevacizumab ≈ 4 mg/kg	Cetuximab	−3 days	From 1 to 13 days		↑ cetuximab		
U87MG	Glioblastoma	Bevacizumab 10 mg/kg	Temozolomide, irinotecan	−7 days	1 h		=/↑ temozolomide =/↑ irinotecan	HPLC	[71]
				0 day			=/↑ temozolomide =/↑ irinotecan		
KPL4	Breast cancer	Bevacizumab 10 mg/kg	Trastuzumab	−7 days	6 h	↓ vessel density	↓ trastuzumab	multispectral ultra FM	[72]
				−3 days		↓ vessel density	↓ trastuzumab		
				−1 day		↓ vessel density	↓ trastuzumab		
KPL4	Breast cancer	B20.4.1 10 mg/kg	Trastuzumab	−1 day	From 1 to 7 days	↓ vessel density ↓ perfusion	↓ trastuzumab	SPECT	[73]
MMTV-HER2 Fo5	Breast cancer	B20.4.1 10 mg/kg	Trastuzumab	−1 day	From 1 to 7 days		↓ trastuzumab		
SUM149	Colon cancer	Bevacizumab 10 mg/kg	Cetuximab, R1507 (Ab a IGF1R)	−4 days	3 days	↓ vessel density	↓ R1507 ↓ cetuximab	SPECT	[74]
SKBR3	Breast cancer	Bevacizumab 10 mg/kg	R1507 (Ab a IGF1R)	−4 days	3 days		↓ R1507		
MDA-MB-435	Breast cancer	A461 1 mg/kg	Cisplatin + 5-FU	−1 day	1 h	↓ perfusion/permeability ↓ vessel density ↑ pericyte coverage	↓ cisplatin = 5-FU	ICP-AES (cisplatin) HPLC (5-FU)	75]
SKOV-3	Ovarian cancer	Bevacizumab 5 mg/kg	Trastuzumab	−1, 2, and 5 days	1 and 6 days		↓ trastuzumab	PET	[76]
OE19	Esophageal cancer	Bevacizumab 5 mg/kg	Trastuzumab	−1, 2, and 5 days	1 and 6 days		↓ trastuzumab		
A2780-1A9	Ovarian cancer	Bevacizumab ≈ 6 mg/kg	Paclitaxel	−5 and −1 days	From 0.25 h to 3 days	↓ vessel diameter ↓ perfusion/permeability ↓ necrosis	↓ paclitaxel	HPLC MALDI	[77]
IGROV1	Ovarian cancer	Bevacizumab ≈6 mg/kg	Paclitaxel	−5 and −1 days	6 h	↓ vessel diameter	= paclitaxel		
HT-29	Colon cancer	Bevacizumab ≈6 mg/kg	Paclitaxel	−5 and −1 days	6 h	↓ vessel density	= paclitaxel		
HT29	Colon cancer	A461 ≈ 8 mg/kg	Irinotecan	−7 and −3 days	1 h	↓ vessel number/diameter ↑ perfusion/permeability = necrosis	=/↑ irinotecan = SN-38 metabolite	HPLC	[78]

= equal; ↓ reduction; ↑ increase; =/↓ no significant reduction; =/↑ no significant increase; *HPLC* high performance liquid chromatography; *IHC* immunohistochemistry; *BPA* p-boronophenylalanine; *PGA* prompt γ-ray spectrometry; *FM* fluorescence microscopy; *SPECT* single photon emission computerized tomography; *ICP-AES* inductively coupled plasma atomic emission spectrometry; *5-FU* 5-fluorouracil; *PET* positron emission tomography; *MALDI* matrix-assisted laser desorption/ionization

We and others have shown that the concentrations of small molecules (cisplatin [75], paclitaxel [77], or doxorubicin [77]) in tumors were decreased after bevacizumab treatment. This decrease was confirmed in different tumor models and with angiogenesis inhibitors (e.g., RTKIs) other than bevacizumab [73, 79, 80]. In our studies, this decrease was often associated with delayed efflux of chemotherapeutics from tumors [77–79]. The reduced uptake of chemotherapeutics after bevacizumab treatment was corroborated by the reduction of tumor perfusion or vessel permeability, as measured by dynamic contrast enhancement-magnetic resonance imaging (DCE-MRI) [77, 81]. Nevertheless, in all models the combination delayed tumor growth significantly more than single treatment. Thus, one could speculate that angiogenesis inhibitors enhance the efficacy of certain chemotherapeutics by prolonging contact time of drugs with neoplastic cells [3, 79]. Some studies clearly illustrate the importance of the treatment schedule, showing the temporary time window in which the antiangiogenic agent exerts beneficial effects on drug pharmacokinetics. In fact, drug penetration in tumors was enhanced only when the chemotherapeutic agent was administered within a narrow interval after anti-VEGF therapy (i.e., bevacizumab) [20, 67, 68].

Most of the pharmacokinetic studies in the clinical literature assessed the concentrations of drugs and their metabolites in plasma but not in the tumor. However, the association between the two compartments may not be direct [82]. To our knowledge, only one study in humans describes the effect of antiangiogenic therapy on chemotherapeutic levels in tumors. It was reported that bevacizumab induced rapid, significant reductions in perfusion and [^11^C]docetaxel uptake in NSCLC [83]. This study highlights the importance of drug scheduling and calls for further analysis to optimize combination modalities.

Outcomes differed in relation to the type and molecular weight of the antitumor drug administered after antiangiogenics. Antiangiogenic therapy can improve nanoparticle uptake in a size-dependent manner, with this effect being limited to drugs with a diameter shorter than 10 nm, whereas the tissue penetration of larger molecules (with a diameter longer than 100 nm) is prevented [84]. In line with this situation, preclinical studies have shown that the pre-administration of anti-VEGF reduces the intratumoral accumulation of therapeutic antibodies [72–74, 76] and control IgG [76], along with the reductions of tumor blood flow and vessel density.

## Effect of anti-VEGF therapy on intratumoral perfusion and drug spatial distribution

Solid tumors are heterogeneous, not only in terms of cancer cell genotype and phenotype but also in their stromal composition. The tumor microenvironment can physically hinder the penetration of chemotherapy to neoplastic tissue, and inadequate arrival of the effective drug to some cancer cells may cause recurrence or limit the response [85].

An extension of the “normalization” theory supports the idea that hemodynamic changes induced by antiangiogenics lead to more uniform distribution of blood flow and to a reduction of hypoxic/necrotic areas in tumor tissue. This situation would favor more homogeneous intratumoral distribution of anticancer therapies. Our understanding of how antiangiogenic pretreatment affects intratumoral distribution of chemotherapeutic agents is far from complete, since experimental data are scanty. Some imaging techniques have been employed to investigate drug localization in tumor tissue, such as positron emission tomography (PET), single photon emission computed tomography (SPECT), magnetic resonance spectroscopy, autoradiography, fluorescence microscopy, and mass spectrometry imaging (MSI) [82, 86]. In an orthotopic neuroblastoma xenograft model, contrast-enhanced ultrasonography indicated that bevacizumab pretreatment induced more homogeneous contrast enhancement throughout the tumor mass than in controls where enhancement was restricted to the tumor periphery [20]. Accordingly, using longitudinal perfusion computed tomography (CT), sorafenib was shown to favor perfusion in areas that initially showed minimal or no blood flow [87]. A clinical study on hepatocellular carcinoma reported that patients in whom bevacizumab reduced tumor blood flow heterogeneity had a better prognosis [88].

Using histological staining and MSI to visualize paclitaxel localization in tissues, we found that its distribution was inadequate in poorly vascularized areas of tumors, but more homogeneous in the bevacizumab-treated tumors, where there was a reduction of necrotic areas and more functional vascularization [77]. This was observed in different tumor xenografts (ovarian and colon), implanted in different (orthotopic and ectopic) sites, and always associated with not increased paclitaxel concentrations. We had similar results, not only after antiangiogenics but also after chronic pretreatment with low doses of paclitaxel, whose antiangiogenic effect was clearly demonstrated [89], favoring homogeneous intratumoral distribution of a single subsequent high dose of paclitaxel [90]. The improved distribution of paclitaxel in tumor tissue might partly explain the antitumor potentiation of the combination with antiangiogenic treatment in solid tumors.

Different results were obtained combining anticancer antibodies with antiangiogenics. A preclinical study using multispectral fluorescence indicated that bevacizumab significantly hampered the penetration of trastuzumab (anti-HER2/neu receptor antibody)-Alexa750 in tumor tissue, despite a more uniform tumor vasculature [72]. The antibody localized solely in the periphery of the bevacizumab-pretreated tumors. Similarly, Pastuskovas et al. [73] showed that anti-VEGF restricted trastuzumab distribution to the tumor margin, consistently with vessel localization. In RIP-Tag2 transgenic mice, the inhibition of VEGF signaling reduced the tumor vascularity. The antibody distribution per surviving tumor vessel was better. Antibodies given after antiangiogenics preferentially accumulate in the sleeves of basement membrane left behind by regressing tumor vessels [91].

## Conclusions

VEGF inhibitors are mainly used in oncology with chemotherapy, but the mechanism by which antiangiogenic agents help chemotherapy is not completely understood. The outcome of using bevacizumab in combination with chemotherapy probably depends on the tumor type and stage and very closely on the dose/schedule of treatment.

It is widely recognized that vessel changes occur after antiangiogenic treatment, but how this can modify vessel patency, TIFP, hypoxia, and ultimately drug uptake and distribution is still not clear. Some preclinical studies reported functional improvement in tumor blood perfusion after angiogenesis inhibitors, with increased tumor exposure to cytotoxic drugs. However, in other studies, tumor vascular patency decreased and hypoxia increased, with impaired cytotoxic drug uptake. The causal relationships between the effect on the microvasculature, the TIFP reduction, and the trans-vascular transport of drugs are still not completely understood.

We have proposed that the greater antitumor activity of paclitaxel after bevacizumab is not necessarily due to high drug concentrations, but to the restoration of a more functional tumor microenvironment that facilitates the distribution of chemotherapy. Whether this holds true for other combination modalities, different angiogenesis inhibitors, and other chemotherapeutics remains to be established. It also needs to be shown whether this paradigm can be translated to patients’ tumors under treatment. Monitoring the activity of antiangiogenics is a practical challenge in the clinical setting, where non-invasive imaging procedures need to be improved to monitor the administration and determine the efficacy of antiangiogenesis-based combination regimens in every tumor.
